# Maternal high-fat diet and obesity compromise fetal hematopoiesis

**DOI:** 10.1016/j.molmet.2014.11.001

**Published:** 2014-11-18

**Authors:** Ashley N. Kamimae-Lanning, Stephanie M. Krasnow, Natalya A. Goloviznina, Xinxia Zhu, Quinn R. Roth-Carter, Peter R. Levasseur, Sophia Jeng, Shannon K. McWeeney, Peter Kurre, Daniel L. Marks

**Affiliations:** 1 Papé Family Pediatric Research Institute, Oregon Health & Science University, Portland, OR 97239, USA; 2 Oregon Stem Cell Center, Oregon Health & Science University, Portland, OR 97239, USA; 3 Department of Pediatrics, Oregon Health & Science University, Portland, OR 97239, USA; 4 Department of Cell & Developmental Biology, Oregon Health & Science University, Portland, OR 97239, USA; 5 Pulmonary & Critical Care, Oregon Health & Science University, Portland, OR 97239, USA; 6 Oregon Clinical and Translational Research Institute, Oregon Health & Science University, Portland, OR 97239, USA; 7 Division of Bioinformatics and Computational Biology, Department of Medical Informatics & Clinical Epidemiology, Oregon Health & Science University, Portland, OR 97239, USA

**Keywords:** Developmental programming, Hematopoietic stem and progenitor cells, Hematopoiesis, High-fat diet, Obesity, Fetal liver

## Abstract

**Objective:**

Recent evidence indicates that the adult hematopoietic system is susceptible to diet-induced lineage skewing. It is not known whether the developing hematopoietic system is subject to metabolic programming via *in utero* high-fat diet (HFD) exposure, an established mechanism of adult disease in several organ systems. We previously reported substantial losses in offspring liver size with prenatal HFD. As the liver is the main hematopoietic organ in the fetus, we asked whether the developmental expansion of the hematopoietic stem and progenitor cell (HSPC) pool is compromised by prenatal HFD and/or maternal obesity.

**Methods:**

We used quantitative assays, progenitor colony formation, flow cytometry, transplantation, and gene expression assays with a series of dietary manipulations to test the effects of gestational high-fat diet and maternal obesity on the day 14.5 fetal liver hematopoietic system.

**Results:**

Maternal obesity, particularly when paired with gestational HFD, restricts physiological expansion of fetal HSPCs while promoting the opposing cell fate of differentiation. Importantly, these effects are only partially ameliorated by gestational dietary adjustments for obese dams. Competitive transplantation reveals compromised repopulation and myeloid-biased differentiation of HFD-programmed HSPCs to be a niche-dependent defect, apparent in HFD-conditioned male recipients. Fetal HSPC deficiencies coincide with perturbations in genes regulating metabolism, immune and inflammatory processes, and stress response, along with downregulation of genes critical for hematopoietic stem cell self-renewal and activation of pathways regulating cell migration.

**Conclusions:**

Our data reveal a previously unrecognized susceptibility to nutritional and metabolic developmental programming in the fetal HSPC compartment, which is a partially reversible and microenvironment-dependent defect perturbing stem and progenitor cell expansion and hematopoietic lineage commitment.

## 1 Introduction

The rise in obesity rates over the past several decades coincides with an increased disease burden in obese individuals and their children. Accumulating epidemiologic and experimental evidence strongly suggests maternal obesity and improper prenatal nutrition provide maladaptive intrauterine cues to developing offspring, ultimately programming organs for predisposition to chronic disease later in life [1,2]. Several studies point to developmental origins of postnatal neurological, cardiovascular and endocrine complications via maternal high-fat diet (HFD), a simplified model of the western-style diet, in the absence of gross organ compromise during infancy [3–7]. These multiple lines of evidence present prenatal development as a period of global susceptibility for diet-induced metabolic injury, fetal programming and postnatal organ dysfunction [8], but it remains unclear as to what extent the developing hematopoietic system is vulnerable.

Adverse developmental programming predisposes individuals to chronic conditions, such as metabolic syndrome [9,10], in which inflammation plays a substantial role [7,11], yet no studies have addressed the impact of fetal programming on the hematopoietic stem cells (HSC) from which adaptive and innate immunity arise. HSCs rely heavily on glycolysis and fatty acid oxidation, and the requirement for them to switch between metabolic states for cell fate decisions—quiescence, self-renewal, or differentiation—leaves them susceptible to nutritional perturbations [12]. HSCs first emerge in the early embryo and go on to rapidly expand in the fetal liver before transitioning to the bone marrow late during gestation [13]. Here, we expand our previous observations that maternal overnutrition severely stunts liver size [14] by asking if the fetal hematopoietic stem and progenitor cell (HSPC) pool is sensitive to metabolic injury.

We report that HFD and maternal obesity perturb both the expansion of the fetal HSPC pool and hematopoietic lineage specification, using the C57BL/6 mouse model of diet-induced obesity. These studies demonstrate a link between developmental programming and canonical regulation of self-renewal and cell migration, known to constrain HSPC function. Our observations of maternal diet- and obesity-induced vulnerability of the fetal HSPC pool provide novel mechanistic and functional evidence for *in utero* metabolic programming of the hematopoietic system.

## 2 Materials and methods

### 2.1 Mice

Animals were handled in accordance with OHSU IACUC. For HFD studies, C57BL/6 CD45.2 female mice (Jackson Labs) were fed a 60% kcal% fat diet (D12492, Research Diets, New Brunswick, NJ) or a 13.5% kcal% fat, control diet (Laboratory Rodent Diet 5001, Lab Diet, St. Louis, MO) *ad libitum*. Acute HFD mice were fed HFD for 2 weeks starting at 9–11 weeks of age and age-matched controls were kept on control diet; both groups were kept on respective diets through breeder pairing and pregnancy. Chronic HFD female mice were fed HFD starting at 5–7 weeks of age, and together with corresponding control diet mice, were sacrificed for fetal harvests at 26–37 weeks of age; HFD-induced obesity but not overt diabetes occurs in these animals. For DR experiments, mice from the chronic HFD cohort were switched to the control diet at 42 weeks of age, bred, kept on control diet through gestation (age-matched controls remained on control diet), and sacrificed at 45–49 weeks of age for fetal harvests. Males were only fed HFD when paired with females for breeding. Pregnancies were timed using the vaginal plug method; fetuses were harvested at day 14.5 of gestation and livers were dissected and prepared in single cell suspensions by pipetting. Cells were counted by hemacytometer or Bio-Rad TC10. Masses were collected as wet weights.

### 2.2 Cell culture

Unfractionated fetal liver cells were plated in mouse methylcellulose complete media (R&D Systems, Minneapolis, MN) at 20,000 cells/mL and performed according to manufacturer's instructions.

### 2.3 Flow cytometry

Cells were prepared from fetal liver and adult bone marrow. The following antibodies were used for analysis: CD3, CD4, CD5, B220, Gr-1, Ter119, c-Kit/CD117, Mac-1, CD45.2 (BD, Franklin Lakes, NJ), CD45.1, Sca-1, AA4.1/CD93, F4/80 (eBioscience, San Diego, CA). Staining reagents were also used for analysis: LIVE/DEAD Fixable Dead Cell Stain (Invitrogen, Carlsbad, CA), propidium iodide (Sigma–Aldrich, St. Louis, MO), Annexin V, and the reactive oxygen species dye carboxy-H_2_DCFDA (Molecular Probes, Eugene, OR). Cells were analyzed on a BD LSR II and a BD FACS Calibur.

### 2.4 Quantitative real-time PCR

For qRT-PCR, HFD or control fetal liver cells were immunomagnetically enriched using the Sca-1 antibody conjugated to PE (clone E13-161.7; BD, Franklin Lakes, NJ) and the EasySep PE Positive Selection Kit (StemCell Technologies, Vancouver, BC) [15]. RNA was extracted with an RNeasy Mini Kit (Qiagen, Valencia, CA), cDNA synthesis was performed with SuperScript III First-Strand (Invitrogen), and reactions were run on StepOnePlus (Applied Biosystems, Foster City, CA). Primers were previously described for *Bmi1* [16], *Hmga2* [17], *Igf2bp2*, *Lin28* [18], and *Mmp9* [19], and normalized to *β-actin*. Primer sequences for murine *Mmp8* and *Egr1* are in Appendix Table A.1. Data was analyzed by using the comparative C_T_ method. Statistics were performed on the relative fold change (2^−ΔΔCT^) of each sample, compared to the mean of the respective control cohort.

### 2.5 Statistical analysis

Means are presented in bar graphs and scatter plots, ±standard error of the mean (error bars), and compared using two-tailed, unpaired Student's *t*-test; a *P* value of 0.05 or less is considered significant. Where appropriate, the false discovery rate is calculated and the corrected significance values (*q*) are presented [20].

### 2.6 RNA-sequencing and analysis

A cohort of dams were fed HFD or control diet for 12–14 weeks, and their offspring were harvested at 15 ± 0.5 dpc; whole livers from 6 control and 6 HFD male fetuses were frozen in RNALater (Qiagen). Total RNA was extracted using RNeasy (Qiagen). An mRNA library was prepared by the OHSU Massively Parallel Sequencing Shared Resource, using the Illumina TruSeq RNA Sample Prep Kit v2. Starting with total RNA, mRNA was purified using polyA selection, then chemically fragmented and converted into single-stranded cDNA using random hexamer priming. Next, the second strand was generated to create double-stranded cDNA. Blunt-end DNA fragments were then generated using fill-in reactions and exonuclease activity. An ‘A’-base was added to the blunt ends of each strand, preparing them for ligation to the coded sequencing adapters. Once the adapters were ligated, the constructs were then subject to 10 rounds of PCR. The amplified libraries were purified and underwent quantitative PCR with an ABI StepOne real-time PCR system. Libraries are diluted to an empirically determined concentration appropriate for the flow cell in use and applied using an Illumina cBot. Flow cells were sequenced on an Illumina HiSeq 2000.

We assessed read quality using fastqc (http://www.bioinformatics.babraham.ac.uk/projects/fastqc/) metrics and aligned to the mm10 genome using Subread 1.3.6-p1 [21]. The biomaRt package from Bioconductor [22] was used for annotation. EdgeR was used to determine differentially expressed genes [23]. *P*-values were adjusted for multiple testing using the Benjamini–Hochberg method [20]. Differentially expressed genes were further filtered by coefficient of variation (CV), calculated as the standard deviation divided by the mean; a gene passed the CV filter if the CV for both the control group and HFD group was less than 2. Processes of interest in which significantly differentially expressed genes are involved were identified by GO Terms from Bioconductor. The data are accessible in the NCBI Gene Expression Omnibus (GEO) through accession number GSE62715 (http://www.ncbi.nlm.nih.gov/geo/query/acc.cgi?acc=GSE62715).

### 2.7 Transplantation

For non-competitive transplantation, CD45.2 females were placed on HFD or control diet at 7–10 weeks of age, and after 20–23 weeks, were bred for timed pregnancies. On the day of fetal harvest (14.5 dpc), 8–13 week-old male CD45.1 mice were irradiated in a cesium irradiator (J.L. Shepherd) with 750 cGy and approximately 7 h later, were each injected with 1 × 10^6^ whole fetal liver cells. Each recipient received a different fetal liver, for a total of 5 chronic HFD and 5 control grafts. Retroorbital blood draws were performed at 4 and 14 weeks post-transplant and peripheral blood was stained with antibodies against CD45.1 and CD45.2 for analysis.

For competitive transplantation, CD45.2 female mice were placed on a HFD starting at 6 weeks of age and were kept on the diet during breeding and pregnancy; they were bred to CD45.1 males and were on the diet for a total of 20–24 weeks; age-matched CD45.2 female mice on control diet were bred to CD45.2 males. Donor fetal livers were harvested at 14.5 dpc and single-cell suspensions were cryopreserved in Iscove's Modified Dulbecco's Medium (Invitrogen, Carlsbad, CA) with 10% FBS, 1% penicillin/streptomycin, and 10% DMSO. For each recipient cohort, five male and five female CD45.1 mice were placed on either a HFD or control diet, starting at 3–7 weeks of age and after 11 weeks, were irradiated with a split dose of 900 cGy (the day prior to transplant). For each graft, fetal liver cells were thawed, washed, and 2.5 × 10^5^ HFD fetal liver cells were mixed with an equivalent number of control fetal liver cells, for a total of 10 different pairs of grafts prepared in duplicate, and were transplanted via tail vein injection into pairs of sex-matched mice on differential diets (also maintained post-transplant). Blood draws and analysis were performed as before.

## 3 Results

### 3.1 Weight gain in high-fat diet-fed and obese pregnant mice

**Figure A.1:**
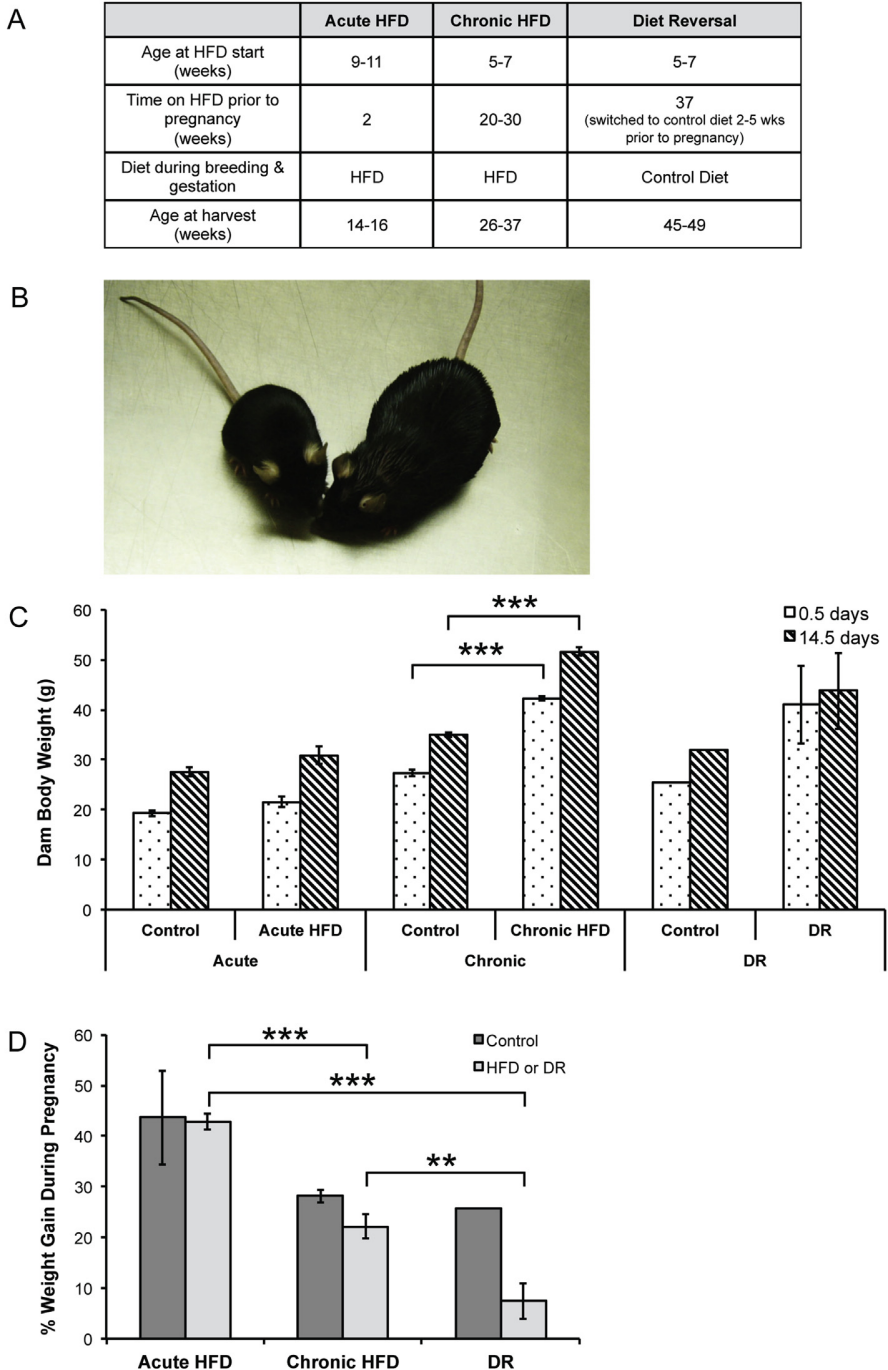
Dietary schematics and maternal weight gain during pregnancy. (A) Summary of dietary schematics and timeline for breeding. Control mice for each respective group were age-matched. (B) Representative photo of a non-pregnant control diet-fed (left) versus a chronic HFD-fed (right) female mouse. (C) Weights for dams on day 0.5 and 14.5 of gestation (*n*_Control_ = 3, *n*_acute_ = 5; *n*_Control_ = 4, *n*_chronic_ = 5; *n*_Control_ = 2, *n*_DR_ = 3). (D) Percent weight gain during pregnancy (expressed as percent of weight by 14.5 dpc to starting weight on gestational day 0.5) was calculated in order to compare dam weight gain across experiments, as the three experimental setups each contained dams at different ages (acute HFD *n*_Control_ = 3, *n*_HFD_ = 5; chronic HFD *n*_Control_ = 4, *n*_HFD_ = 5; DR *n*_Control_ = 2, *n*_DR_ = 3). Error bars are standard error of the mean. Asterisks indicate *P* ≤0.05 for *, *P* ≤0.01 for **, and *P* ≤0.001 for ***.

To test the effects of a lipid-rich diet and/or obesity on fetal hematopoiesis, we used three different dietary strategies: a HFD alone, a HFD with maternal obesity, and obesity without HFD (Figure A.1A). We monitored weight gain from 0.5 to 14.5 dpc (days post coitum) in dams for all three sets. A representative image of a non-pregnant control and obese female mouse is shown in Figure (A.1B). Compared to controls, chronic HFD dams had significantly higher body masses at both 0.5 and 14.5 dpc (Figure A.1C). We also calculated the percent weight gain of dams during gestation in order to compare different treatments; acute HFD dams gained significantly more than obese dams, as they began gestation with a lower body mass (Figure A.1D).

### 3.2 Acute HFD boosts growth and promotes hematopoietic expansion and differentiation in the fetal liver

**Figure 1:**
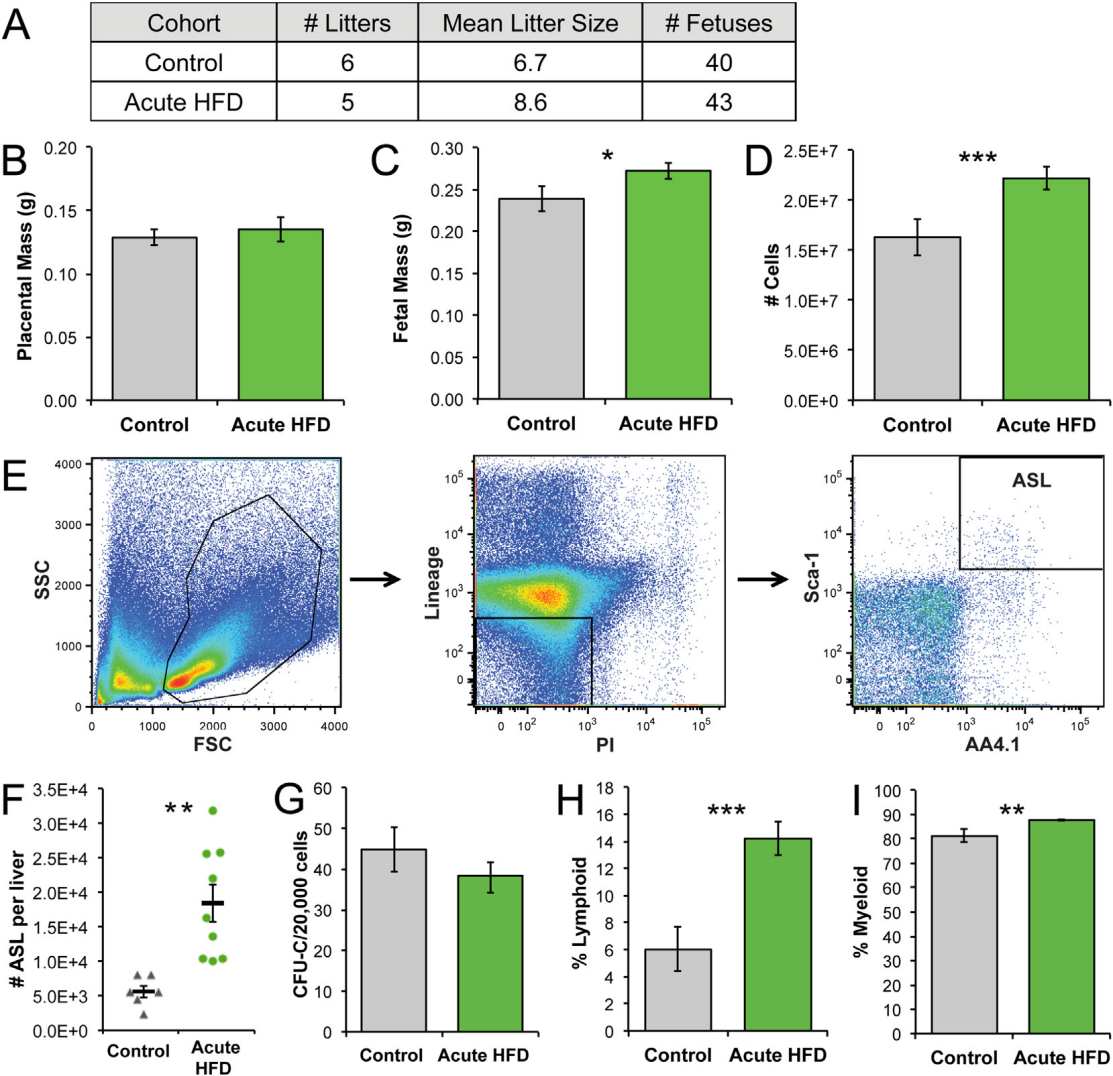
Acute HFD induces a significant increase in fetal liver hematopoietic cellularity. (A) Litter data. (B) Placental weights. (C) Fetal weights. (D) Cells per liver. For B–D, *n*_Control_ = 40, *n*_HFD_ = 43. (E) Flow cytometry gating of fetal liver cells, viable lineage (CD3, CD4, CD5, B220, Gr-1, Ter119) low or negative cells, and ASL cells. (F) The number of ASL HSPCs per liver was calculated from flow cytometry and liver cell counts, *n*_Control_ = 6, *n*_HFD_ = 9. (G) Hematopoietic progenitor cell frequency as colony forming units per 20,000 cells, *n*_Control_ = 10, *n*_HFD_ = 8. (H) Percent lymphoid (B220^+^/CD3^+^) cells in fetal liver. (I) Percent myeloid (Gr-1^+^/Ter119^+^) cells in fetal liver. Error bars reflect standard error of the mean; asterisks indicate *P* ≤0.05 for *, *P* ≤0.01 for **, and *P* ≤0.001 for ***.

We investigated the effect of HFD without preexisting obesity on fetal hematopoietic programming by first feeding HFD or control diet to a cohort of female mice for 2 weeks to allow the animals to adjust to the new diet without becoming obese. Each diet was continued during subsequent mating and gestation until fetal harvest at 14.5 dpc. We consistently observed that acute HFD litter sizes were larger than controls (Figure 1A, *P* = 0.02). Both the body mass and liver cellularity of HFD fetuses were significantly increased relative to controls, while the placental masses of HFD fetuses were approximately equivalent to controls (Figure 1B–D). The number of HSPCs within the AA4.1^+^ Sca-1^+^ Lin^low/−^ (ASL) population was increased in acute HFD fetal livers (Figure 1E–F), but the hematopoietic progenitor cell (HPC) frequency by colony formation assay was lower than in controls (Figure 1G). Within more differentiated cell subsets, acute HFD fetal livers were comparatively enriched in both lymphoid (CD3^+^/B220^+^) and myeloid (Gr1^+^/Ter119^+^) cells, relative to controls (Figure 1H–I). In sum, acute HFD is sufficient to increase fetal body mass, liver cellularity, and expansion of the ASL HSPC population as well as the lymphoid and myeloid lineages in the fetal liver.

### 3.3 Maternal obesity and HFD significantly stunt the fetal liver stem and progenitor cell pool

**Figure 2:**
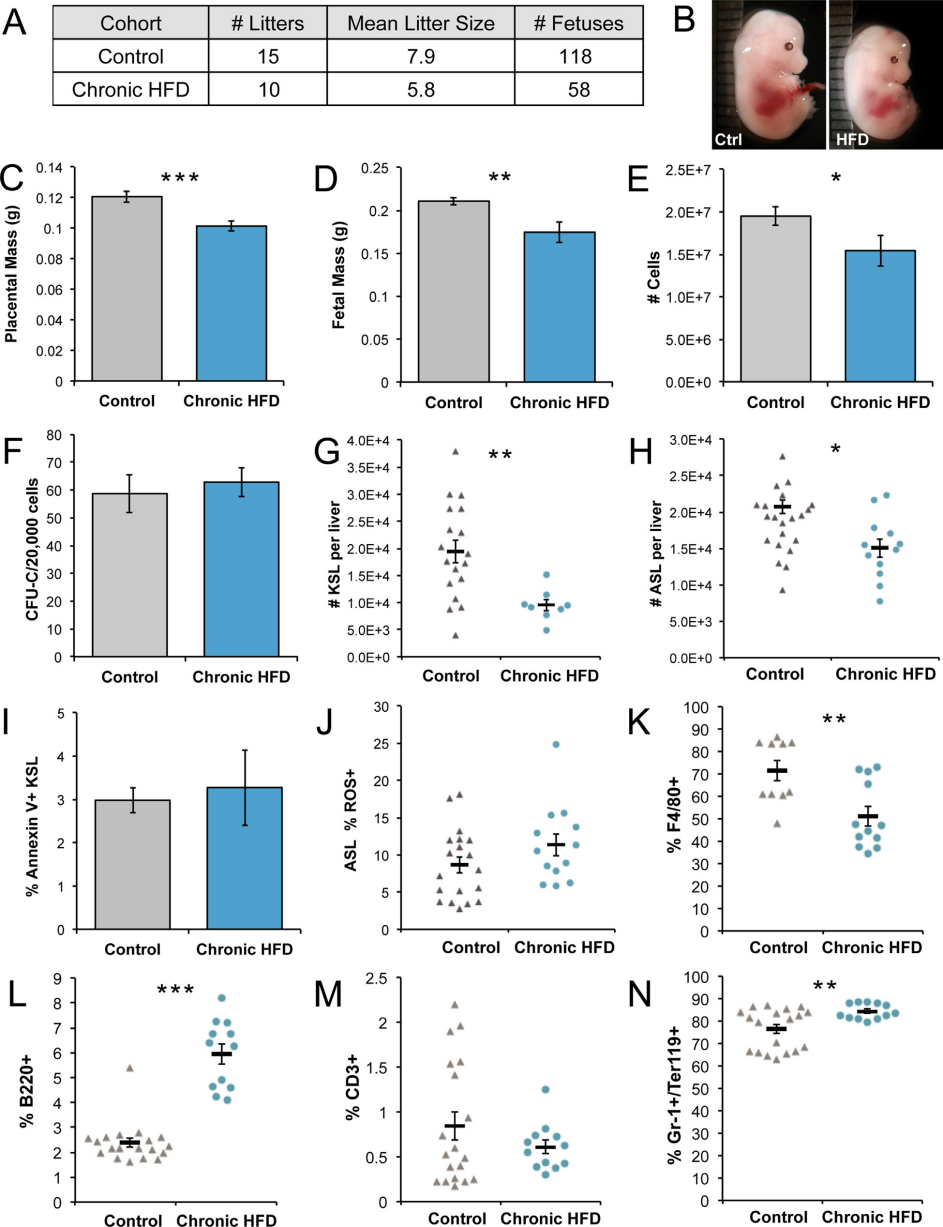
Maternal obesity with HFD causes a significant decrease in fetal liver HSPCs. (A) Litter data. (B) Representative photo of control and chronic HFD fetal mice. (C) Placental weights (*n*_Control_ = 32, *n*_HFD_ = 27). (D) Fetal weights (*n*_Control_ = 32, *n*_HFD_ = 27). (E) Cells per liver (*n*_Control_ = 55, *n*_HFD_ = 39). (F) Hematopoietic progenitor cell frequency as colony forming units per 20,000 cells. (G) Number of c-Kit^+^ Sca-1^+^ Lin^low/−^ cells in HFD versus control-diet fetal livers (*n*_Control_ = 18, *n*_HFD_ = 8) and (H) ASL cells (*n*_Control_ = 21, *n*_HFD_ = 12). (I) Annexin V^+^ KSL fetal liver cells (*n* = 3 per cohort). (J) Percent of ROS+ ASL cells, measured by staining by the ROS detection dye, carboxy-H_2_DCFDA. (K) CD3^+^, (L) B220^+^, and (M) Gr-1^+^/Ter119^+^ fetal liver cells (N) Percent of F4/80^+^ cells. Error bars reflect standard error of the mean; asterisks indicate *P* ≤0.05 for *, *P* ≤0.01 for **, and *P* ≤0.001 for ***.

We next examined the effect of maternal obesity and HFD on hematopoietic development. Diet-induced obesity occurred in female mice chronically fed a HFD for (Figure A.1C) and their average litter size at day 14.5 of gestation was significantly smaller than that of dams fed a control diet (*P* = 0.03, Figure 2A). Chronic HFD 14.5 dpc placentas and fetal mice were an average of 16% and 17% lighter than controls, respectively (Figure 2B–D), suggesting possible intrauterine growth restriction. The cellularity of chronic HFD fetal livers was diminished by 21%, compared to controls (Figure 2E). Although chronic HFD fetal livers had approximately the same frequency of clonogenic HPCs as controls (Figure 2F), they contained significantly fewer immunophenotypic c-Kit^+^ Sca-1^+^ Lin^low/−^ (KSL) and ASL HSPCs, totaling a 51% decrease and 27% decrease, respectively (Figure 2G–H). We next investigated the possibility that some of the HSPC cell loss is attributable to apoptosis at this time of development using Annexin V and propidium iodide staining followed by flow cytometry analysis, but did not find a difference between the two cohorts (Figure 2I). As others have shown that HFD increases oxidative stress and oxidative DNA damage, to which HSPCs are especially vulnerable [24–26], we also tested for elevated reactive oxygen species in the ASL population, but there was no significant difference between the chronic HFD and control livers (Figure 2J). Differentiation is another alternate cell fate, which could be prioritized over self-renewal to account for HSPC loss in chronic HFD fetal livers. Since erythropoiesis is a major process of the fetal liver, we assayed for liver content of F4/80^+^ cells, which reside at the center of blood islands [27], and found a significant reduction of these cells in the chronic HFD cohort (Figure 2K). Conversely, an increase in the proportion of B220^+^ lymphoid and Gr-1^+^/Ter119^+^ myeloid cells in HFD livers was observed within the blood lineage subsets, although the percentage of CD3^+^ lymphoid cells was comparable to controls (Figure 2L–N). This suggests that chronic HFD may bias fetal livers HSPCs toward myeloid and B cell differentiation at the expense of stem cell self-renewal. In sum, chronic HFD-fed, obese dams were less fertile and their offspring exhibited multiple indications of adverse metabolic programming, including growth retardation at day 14.5 dpc, a decrease in HSPC content in fetal livers, and a concomitant increase in differentiated blood cells.

### 3.4 Diet reversal during pregnancy ameliorates liver cellularity but not HSPC content

**Figure A.2:**
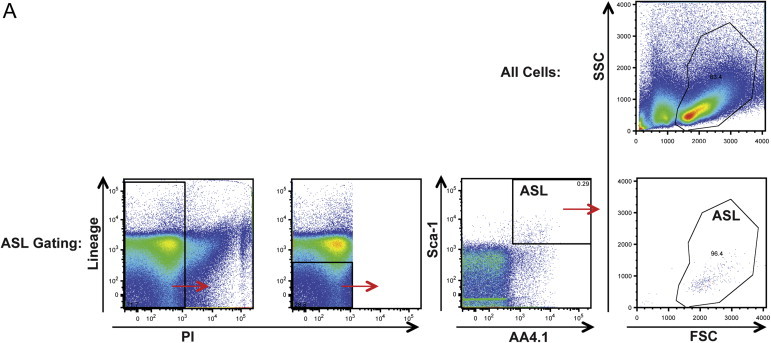
Fetal liver ASL flow cytometry gating validation. (A) Flow cytometry dot plots showing gating of fetal liver cells in forward scatter (FSC) and side scatter (SSC), compared to gating starting with viability and lineage markers, then Sca-1 and AA4.1, and backgating analysis of ASL cells in FSC and SSC.

**Figure 3:**
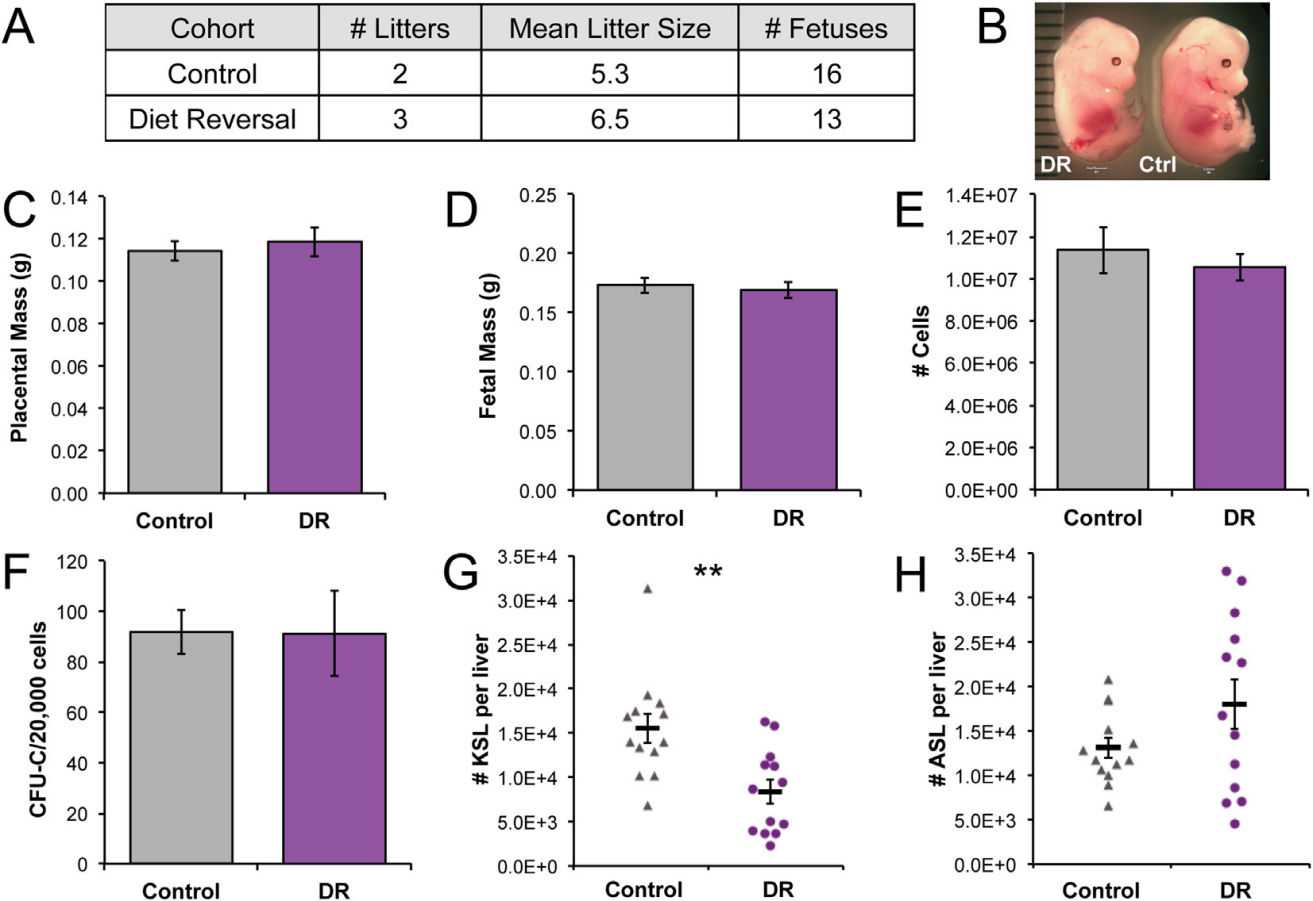
Maternal diet reversal ameliorates some fetal defects, but not diminished HSPC content. (A) Litter data. (B) Photo of DR and control fetuses. (C) Placental weights. (D) Fetal weights. (E) Cells per liver. For C-E, *n*_Control_ = 16, *n*_DR_ = 13. (F) Hematopoietic progenitor frequency, *n*_Control_ = 3, *n*_DR_ = 3. (G) Percent KSL and (H) ASL cells in fetal liver (*n*_Control_ = 13, *n*_DR_ = 13). Asterisks indicate *P* ≤0.05 and *P* ≤0.01 for **. DR, diet reversal.

Our results offer a model in which to test whether the effects of HFD and obesity could be mitigated by a dietary intervention, a key question when extrapolating to human populations. We addressed this possibility by reverting former chronically HFD-fed, obese dams to a control diet during mating and gestation, and then assaying their offspring at 14.5 dpc. Using 3 independent diet reversal (DR) litters, we found that in contrast to the chronic and acute HFD cohorts, there were no significant differences in the placental or body masses of diet reversal (DR) fetal mice, compared to controls (Figure 3A–D), nor were there significant differences in fetal liver cellularity and hematopoietic progenitor cell frequency (Figure 3E–F). Calculation of HSPCs per liver revealed a 46% loss in KSL cells and no significant difference in ASL cell content in DR offspring (Figure 3G–H). There were no significant increases in lymphoid and myeloid populations in DR livers (Figure A.2A–B). These results demonstrate that it is possible to ameliorate the effects of chronic maternal HFD on placental and fetal mass, despite persistence of maternal obesity. Importantly, the fact that DR did not completely normalize liver KSL numbers suggests that it is mainly the obese intrauterine environment that compromises liver HSPC expansion.

### 3.5 Engraftment of HFD-programmed fetal liver cells is constrained by the HFD male microenvironment

**Figure 4:**
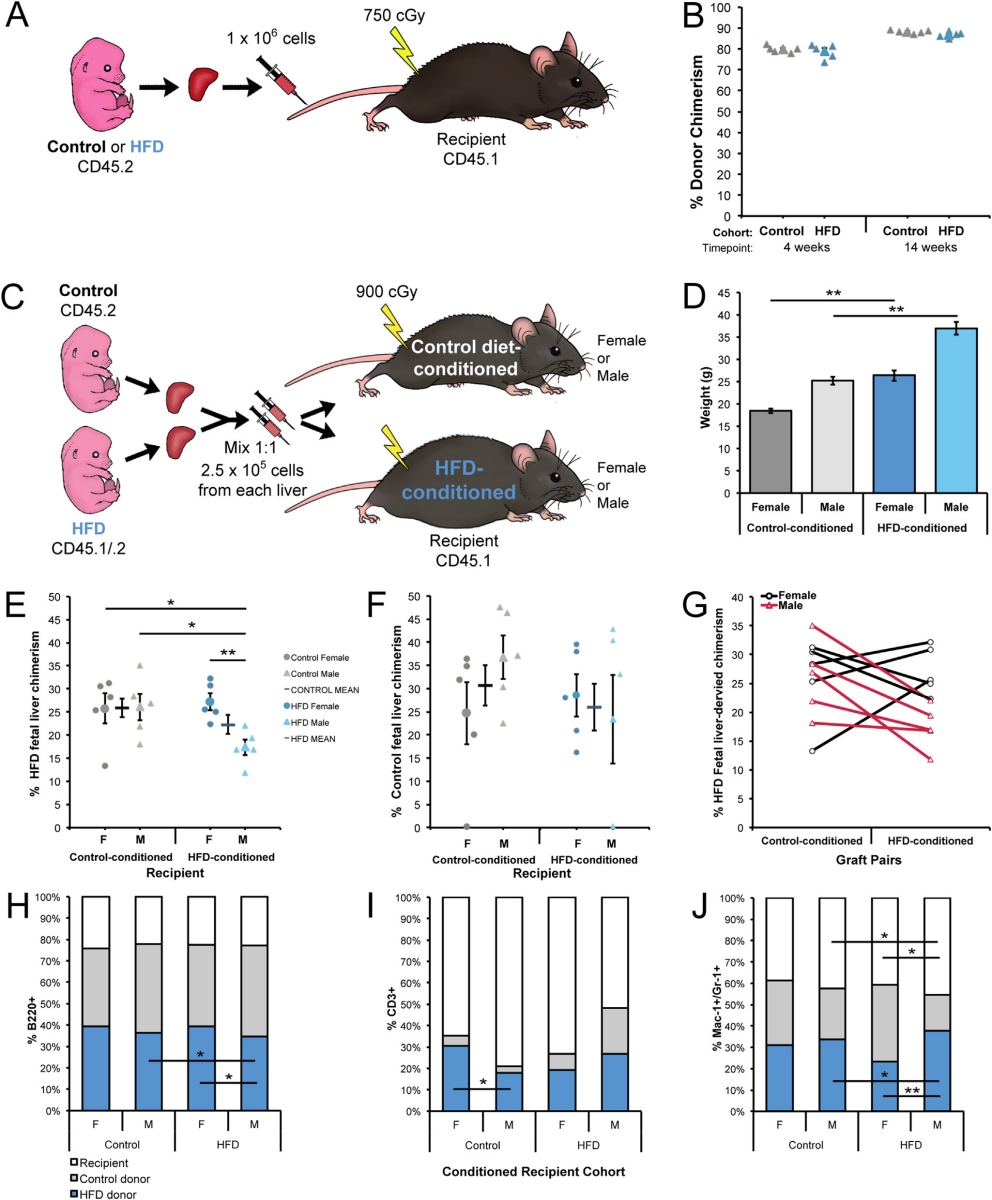
Reduced engraftment of chronic HFD fetal liver cells in the HFD male niche. (A) Schematic for non-competitive transplantation of HFD and control diet fetal liver cells. (B) Peripheral blood fetal liver-derived donor chimerism for non-competitive transplantation at 4 and 14 weeks post-transplant. (C) Schematic for competitive transplantation of HFD versus control fetal liver cells into diet-conditioned recipients. (D) Body weights of recipient mice after 11 weeks of nutritional preconditioning, just prior to transplantation. Peripheral blood chimerism of (E) HFD or (F) control diet fetal liver cells in diet-conditioned recipients at 4 weeks post-transplant. (G) Comparison of HFD fetal liver chimerism in graft pairs; duplicate grafts were given to 2 recipients of the same sex, which were conditioned on different diets. Blood lineage chimerism for residual recipient CD45.1, control donor fetal liver CD45.2, and HFD donor fetal liver CD45.1/.2 cells in (H) B220^+^, (I) CD3^+^, and (J) Mac-1^+^/Gr-1^+^ populations. HFD, high-fat diet; cGy, centigray; F, female; M, male. Error bars reflect standard error of the mean. Asterisks indicate *P* ≤0.05 for * and *P* ≤0.01 for **.

We next investigated the reconstitution capacity of fetal liver HSPCs to determine the functional consequences of HFD programming. Transplantation of CD45.2 chronic HFD or control 14.5 dpc fetal liver cells into irradiated CD45.1 adult male recipients (Figure 4A) yielded comparable levels of engraftment for HFD and control fetal liver cells (Figure 4B). These data, along with the HSPC quantification showing partial amelioration of the fetal phenotype with diet reversal, suggest that removal of the HSPCs from the HFD microenvironment enables their functional recovery, in contrast to the results seen in chronic HFD fetal livers, which had significant deficits in immunophenotypically defined HSPCs. We therefore performed a competitive transplantation of HFD- versus control diet-programmed fetal liver cells into female (F) and male (M) mice conditioned on either a HFD or control diet for 11 weeks prior to transplant and thereafter (Figure 4C). Each graft was prepared in duplicate, with a mix of 2.5 × 10^5^ each of HFD- and control-programmed fetal liver cells, and given to a pair of recipient mice of the same sex, conditioned on different diets (Figure 4C). Dietary conditioning with HFD for 11 weeks prior to transplant resulted in a significant boost in body weight, compared to control diet (Figure 4D). The repopulating capacity of HFD fetal liver was significantly compromised in male HFD mice, compared to all other cohorts (Figure 4E), but the peripheral blood chimerism of control fetal liver cells was not significantly different between any recipient groups (Figure 4F). A comparison of each recipient pair demonstrated a relative suppression of repopulation by HFD-programmed fetal liver in 7 HFD recipients (including all males) compared to their control diet partners, whereas 3 female HFD recipients experienced modestly increased chimerism in relation to their controls (Figure 4G). Male HFD recipients experienced a significant reduction in HFD fetal liver-derived B220^+^ B cells, but only male controls had fewer CD3 T cells compared with female control recipients (Figure 4H–I). By contrast, the male HFD group showed a greater proportion of HFD fetal liver-derived Mac-1^+^/Gr-1^+^ myeloid cells than in female HFD or male controls (Figure 4J). Echoing the partial phenotype correction following *in utero* diet reversal, these data indicate that the HFD fetal HSPC repopulation capacity is diminished in a HFD microenvironment, but can be rescued in a control microenvironment.

### 3.6 Maternal obesity & HFD alter expression of genes involved in stress, hematopoiesis, and stem cell self-renewal & mobilization

**Figure 5:**
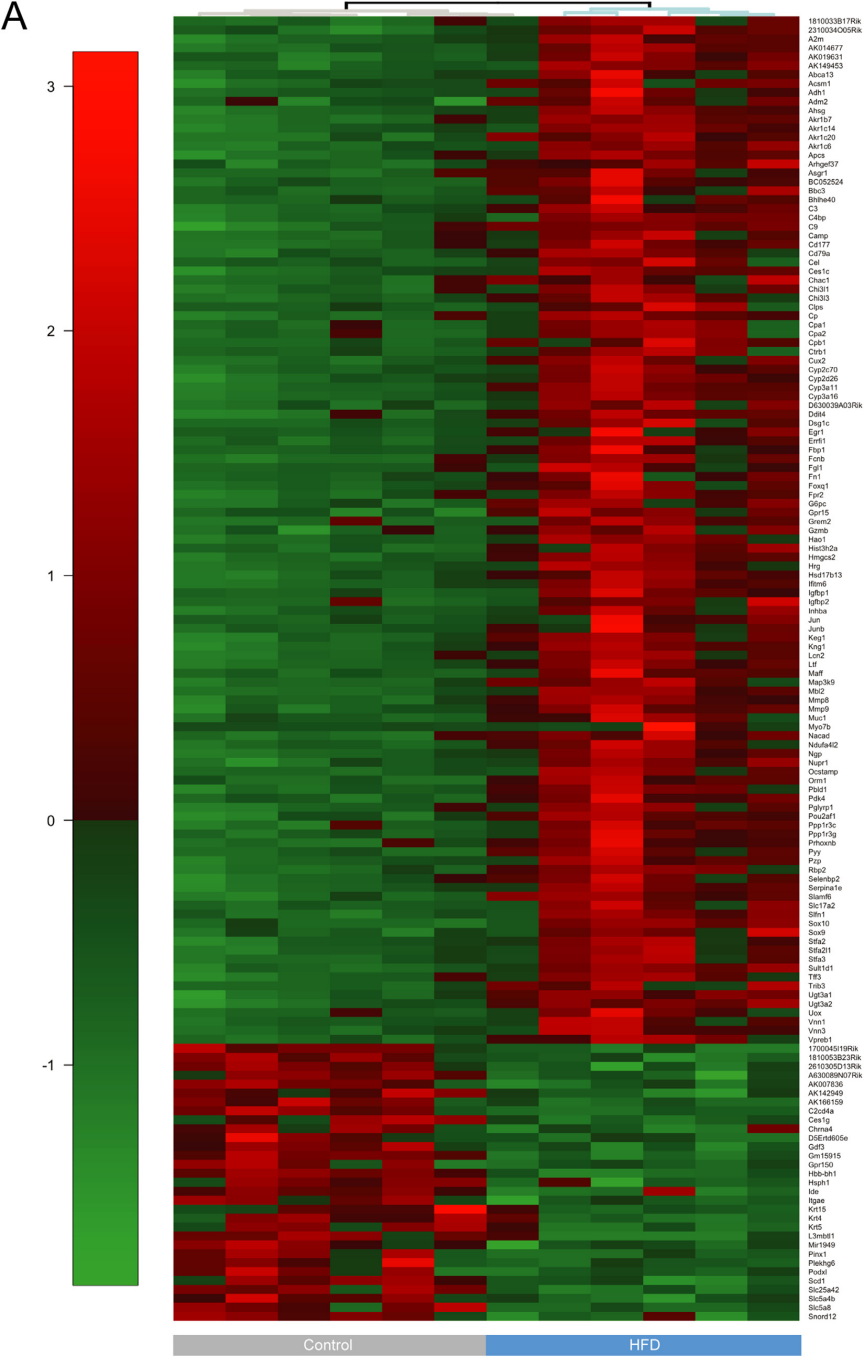
Differential expression of transcripts in HFD versus control fetal livers. (A) Heatmap visualization of differentially expressed* (DE) transcripts between high-fat and control diet programmed, male, 15 ± 0.5 dpc fetal liver cells, as analyzed by RNA-seq. Transcripts with significant fold changes, based on both fold change and FDR adjusted *P*-value threshold, are shown in the heat map. Gene names are indicated to the right of the heat map and cohort is shown at the bottom. Red = upregulation, green = downregulation. Dendrogram indicates sample clustering. *DE genes defined as having an FC >1.5 and FDR <0.05 in both the common and tagwise dispersion estimate analysis.

We expanded on our observations using RNA sequencing (RNA-seq) as an unbiased approach to gain a more comprehensive understanding of the fetal hematopoietic response to maternal HFD. To do this, we analyzed the transcriptome of HFD-programmed versus control fetal livers and found upregulation of 115 genes and downregulation of 31 genes (Figure 5A), in this case using male tissue, in which more pronounced effects are observed [28]. Several of the targets that we identified to be transcriptionally altered have roles in regulation of metabolism, immunity or inflammation, mitochondrial functions, cell migration or adhesion, stress, insulin signaling, hematopoiesis, and other processes (Table 1). The *Early growth response-1* (*Egr-1*) gene, normally expressed at low levels in fetal liver HSC [29] in preparation for HSC migration to the BM compartment [30], was comparatively upregulated in chronic HFD fetal livers, consistent with increased expression of *matrix metalloproteinase 8* (*Mmp8*) and *9* (*Mmp9*), both regulators of HSPC migration [31,32]. Six transcripts involved in complement activation were among the upregulated transcripts in HFD fetal livers; this process is also activated in nonalcoholic fatty liver disease (NAFLD) [33].

**Figure 6:**
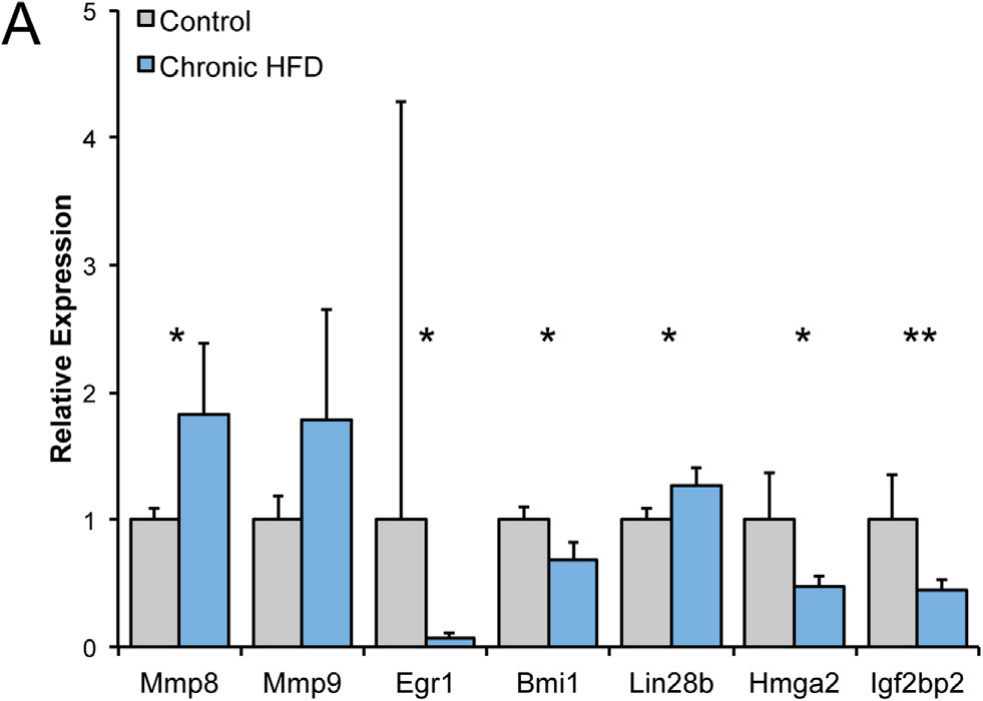
Differential expression of genes involved in self-renewal and migration in HFD and control fetal liver cells. (A) QRT-PCR analysis of Sca-1-enriched chronic HFD versus control fetal liver cells. The corrected significance level (false discovery rate) is *q* = 0.04.

We next immunomagnetically isolated fetal liver cells using Sca-1 as a marker, to determine whether genes of interest identified by RNA-seq in whole livers were differentially expressed within an HSPC-enriched subset of cells. As in RNA-seq, *Mmp8* was upregulated in HFD livers, and *Mmp9* trended upward, though it was not significantly different from controls (Figure 6A). *Egr-1* was expressed at lower levels within the Sca-1-enriched population in chronic HFD, consistent with the results from the matrix metalloproteinases that suggest aberrant egress of HSPCs. Chronic HFD, Sca-1-enriched fetal liver cells also expressed lower levels of *Bmi1*, which has protective functions against ROS and in adult HSC, regulates activation of DNA damage response and is required for self-renewal and maintenance [34,35]. Both *Hmga2* and *Igf2bp2*, which should be richly expressed in highly self-renewing fetal liver HSPCs [18], were downregulated as well, suggesting a reduction in the prenatal expansion of the HSPC compartment. In sum, these gene expression data corroborate a prenatal stress response to HFD and further suggest suppression of HSPC self-renewal and possible premature mobilization during a critical developmental transition for HSPC from the fetal liver to the bone marrow stem cell niche.

## 4 Discussion

Prenatal development is a period of rapid growth and epigenetic remodeling, during which an organism is particularly vulnerable to conditions that adversely impact organ development and disease propensity later in life. Fetal programming of adult disease via maternal nutrition has been revealed in many retrospective studies. Both epidemiological and experimental evidence support a role for maternal obesity and prenatal HFD in reproductive deficiencies, fetal growth restriction, defects in brain development, cardiac abnormalities, and endocrine dysfunction [3–7]. Here, we show for the first time that an adverse metabolic microenvironment during fetal hematopoietic development limits HSPC self-renewal and promotes differentiation.

The skewing of B cell, T cell, and myeloid cell differentiation at such an early stage of development suggests that developmental programming of the immune system by maternal obesity and HFD may affect later immune system response. While we did not analyze the fetal thymus to investigate the main repository of T cell progenitors, both B and T cell and myeloid progenitor cells are present in the fetal liver, concordant with the production of differentiated blood cells that occurs there. It is possible that signaling, such as nutrients or maternal cytokines, could directly or indirectly promote one type of differentiation over another in the context of our fetal model, though further studies are needed in order to assess this. In adult diet-induced obesity mice, loss of B and T cells has been previously reported in males [36], though not in the context of HFD-programmed fetal HSPC engraftment, as seen in our competitive transplantation. In obese humans and animal models, numerous studies have demonstrated chronic low-level inflammation and impairments in immune defense against bacterial and viral infections [37,38]. Fetal immune cells may also be capable of activating to increase the risk of NAFLD, as prenatal onset of hepatic inflammation under HFD and obesity has been demonstrated in non-human primates [39]. Furthermore, the positive correlation between asthma rates and body mass index, insulin resistance, and increased levels of serum triglycerides in children, as well as the link between insulin resistance or diabetes and reduced lung function, lend strong evidence to a potential role for prenatal programming of immune dysfunction [40].

We demonstrate the susceptibility of the fetal liver to prenatal metabolic injury and the subsequent compromise of this critical site for HSPC pool expansion. This work complements our previous studies in neonatal mice and fetal macaques that exhibited a disproportionate restriction of liver growth when gestated under a lipid-rich diet (hematopoietic function was not tested in those studies) [14,41]. Our data also support recent reports that short-term HFD exposure leads to hematopoietic expansion [42]. Substantial amplification of lymphoid cell populations in the acute and chronic HFD groups suggests that prenatal HFD exerts pressure on the fetal hematopoietic system to prioritize lymphocyte development, in line with a study in which 3–6 months of HFD feeding in adult mice resulted in elevated bone marrow lymphocytes and thymic enlargement [43]. In our study, prenatal HFD, compounded by maternal obesity, promoted excess differentiation of both lymphoid and myeloid cell lineages in fetal livers, consistent with a reduction in the opposing cell fate of self-renewal and a recent report that diet-induced obesity can skew adult murine blood differentiation toward myelopoiesis [44]. In adult mice, HFD has been demonstrated to dramatically increase serum leptin levels, especially with weight gain, and this stimulates myelopoiesis and lymphopoiesis [43]. Studies in mice and rats have found leptin and certain leptin receptor isoforms to be expressed in the fetal liver and placenta; with HFD, fetal leptin levels increase [45–48]. Further studies are needed to determine whether leptin signaling plays a role in skewing fetal hematopoiesis under obesity and HFD, though its roles in lymphopoiesis, erythropoiesis, and myelocytic progenitor proliferation have been demonstrated in adults [49,50]. Postnatal HSPC are profoundly susceptible to metabolic cues and rely heavily upon glycolysis and fatty acid oxidation for control of cell fate, and though little is known about fetal HSPC metabolism, a recent study reported that glucose metabolism increases HSC production in zebrafish [12,51]. Glucose and glutamine are also necessary for erythroid and myeloid differentiation, and the availability of both in blood is affected by obesity [52]. Our data indicate that metabolic perturbation of the fetal stem cell compartment, demonstrated in both conditions involving maternal obesity, limits self-renewal and skews lineage specification, whereas acute HFD exposure may simply accelerate normal blood differentiation.

These observations on the impact of diet manipulation on HSPC pool composition are corroborated by our gene expression studies. Metabolic, immune and inflammatory processes were the most common categories encompassing differentially expressed transcripts, particularly upregulated genes, in HFD fetal livers, suggesting broad dysregulation. Within HSPC-enriched chronic HFD fetal liver cells, a fetal lymphoid differentiation regulator, *Lin28b*, was slightly upregulated; although this has been shown to positively regulate *Hmga2*, the expression of both *Hmga2* and a direct target, *Igf2bp2*, were down [18,53], suggesting that this HSPC-specific developmental pathway is dysregulated, contributing to a loss of rapid prenatal expansion. Further, concerted upregulation of both *Matrix metalloproteinase 8* (*Mmp8*) and *9* (*Mmp9*) via HFD suggest premature egress of HSPC from the fetal liver niche [31,32]. Postnatal stress-induced HSPC migration is demonstrated by others, consistent with the notion of niche involvement in the observed fetal HSPC phenotype [54–56].

We show that chronic HFD fetal liver HSPCs are especially sensitive to the unfavorable hematopoietic microenvironment in HFD-conditioned male mice and are perhaps susceptible to the inflammatory milieu that arises particularly in males [57]. Estrogen has a protective effect against obesity and glucose intolerance (in mice) [58], and increases self-renewal of HSCs [59], which may explain why donor chimerism in female recipients was higher than that in males. An exaggerated response to inflammation and the myeloid lineage bias, seen in HFD male recipients, echo an aging phenotype of low-grade inflammation and myeloid-dominant hematopoiesis [60]. Finally, a male predilection has already been shown for adverse developmental programming of hypertension, impaired renal development, placental inflammation, and complications from maternal diabetes [28].

## 5 Conclusions

In summary, our data identify the hematopoietic compartment as a previously unrecognized target for nutritional developmental programming. Metabolic cues impact stem cell development, lineage specification, and both early formation and reconstitution of the blood system. While HFD in the short-term boosts expansion of the fetal hematopoietic compartment, the complex metabolic changes that coincide with obesity put significant constraints on growth and expansion of fetal liver HSPCs. The link between maternal health, prenatal nutrition and childhood diseases involving immune progeny of the HSC compartment [2,8,61,62] highlight the need to better understand the susceptibility of the developing hematopoietic system to metabolic dysregulation, especially in light of the spreading western-style diet and accompanying obesity epidemic.

## Authorship contributions

A.N.K. designed and performed the experiments, wrote and edited the manuscript. N.A.G., and S.M.K. designed and performed the experiments. P.R.L., X.Z. and Q.R.C. performed experiments. S.J. and S.K.M. guided RNA-seq bioinformatics and wrote the corresponding methods. P.K. and D.L.M. designed experiments, and wrote and edited the manuscript.

## Figures and Tables

**Table A.1:** qRT-PCR primer sequences for *Mmp8* and *Egr1*.

Gene	Forward primer	Reverse primer
*Mmp8*	CTT GCC CAT GCC TTT CAA CC	GGG GTT GTC TGA AGG TCC ATA
*Egr1*	ACC TAC ACT CCC CGC AGT GT	AGG ATT GAA GGC GCT GCT GC

**Figure A.3:**
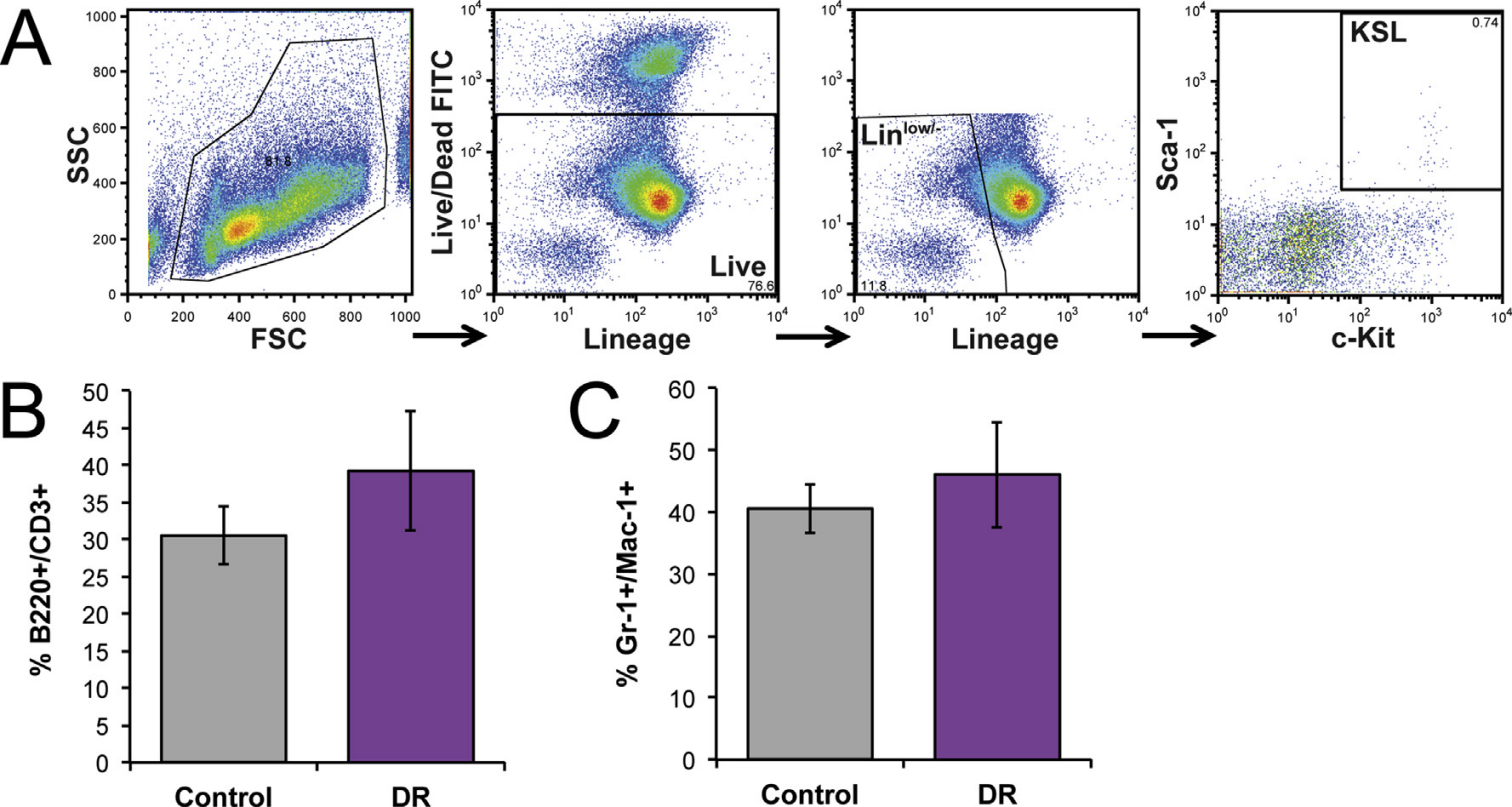
Lymphoid and myeloid fetal liver cells in DR cohort. (A) Flow cytometry gating schematic for fetal liver KSL cells. (B) Percent lymphoid cells and (C) myeloid cells in DR versus control fetal livers.

**Table 1:**
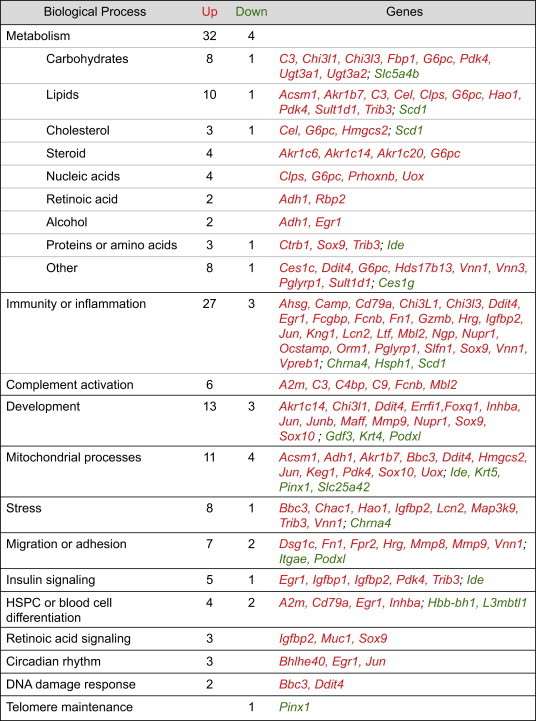
Processes related to differentially expressed transcripts in HFD-programmed fetal liver from RNA-seq. Red = upregulated transcripts, green = downregulated transcripts. FL, fetal liver.
